# Patient preferences for massively parallel sequencing genetic testing of colorectal cancer risk: a discrete choice experiment

**DOI:** 10.1038/s41431-018-0161-z

**Published:** 2018-05-25

**Authors:** Deirdre Weymann, David L. Veenstra, Gail P. Jarvik, Dean A. Regier

**Affiliations:** 1 Canadian Centre for Applied Research in Cancer Control (ARCC), Cancer Control Research, British Columbia Cancer, Vancouver, BC Canada; 2 The Comparative Health Outcomes, Policy & Economics (CHOICE) Institute, Department of Pharmacy, University of Washington, Seattle, WA USA; 3 Department of Medicine (Medical Genetics), University of Washington, Seattle, WA USA; 4 School of Population and Public Health, Faculty of Medicine, University of British Columbia, Vancouver, BC Canada

## Abstract

This study enumerated patients’ preference-based personal utility and willingness-to-pay for massively parallel sequencing (MPS) genetic testing of colorectal cancer (CRC) risk. Our setting was the New Exome Technology in (NEXT) Medicine Study, a randomized control trial of usual care genetic testing vs. exome sequencing. Using a discrete choice experiment (DCE), we elicited patient preferences for information on genetic causes of CRC. We estimated personal utility for the following four attributes: proportion of individuals with a genetic cause of CRC who receive a diagnosis, number of tests used, wait time for results, and cost. A total of 122 patients completed our DCE (66% response rate). On average, patients preferred genetic tests identifying more individuals with a diagnosis and involving a shorter wait time. Assuming MPS identifies more individuals with a Mendelian form of CRC risk, involves fewer tests, and results in a shorter wait than traditional diagnostic testing, average willingness-to-pay (WTP) for MPS ranged from US$400 (95% CI: $300, $500) to US$1541 (95% CI: $1224, $1859). These results indicate that patients value information on genetic causes of CRC and replacing traditional diagnostic testing with MPS testing will increase patients’ utility. Future research exploring the costs and benefits of MPS for CRC risk is warranted.

## Introduction

Colorectal cancer (CRC) is among the most common cancers worldwide and is the fourth leading cause of cancer deaths [1]. An estimated 20 to 30% of CRCs involve a hereditary component, but only ~5% are caused by highly penetrant inherited Mendelian pathogenic variants [2]. Identifying germline causes of inherited CRC offers a number of benefits for patients including confirming diagnoses, refining screening surveillance, and initiating cascade screening for relatives [3, 4]. Individuals who are found to have a pathogenic variant and undergo intensive surveillance through colonoscopy every 1–2 years can reduce their risks of: developing the disease, advanced stage tumors, and death [5]. Yet genetic testing may also lead to inconclusive findings, over-diagnosis, and patient anxiety [4, 6].

The most common Mendelian risk of CRC is due to Lynch syndrome, also known as hereditary nonpolyposis CRC [2]. Lynch syndrome accounts for approximately half of Mendelian CRC cases and currently recommended genetic testing involves a time consuming, multistep process, with 80% sensitivity [7, 8]. This process begins with immunohistochemistry (IHC) and/or microsatellite instability (MSI) tumor testing for a particular protein deficiency associated with a genetic variant. If these assays suggest a deficiency or loss of function in one of the genes known to cause Lynch syndrome (mismatch repair (MMR) genes), a conclusive germline sequencing test then determines whether there is a pathogenic variant present. Even after multiple tests, fewer than half of patients with clinical suspicion of Lynch syndrome have a pathogenic variant detected [9].

As a result of evidence generated by molecular screening initiatives [10–12], current international guidelines recommend that all newly diagnosed CRC patients undergo genetic testing for Lynch syndrome [7, 8, 13, 14]. To detect other less prevalent forms of Mendelian CRC risk, massively parallel sequencing (MPS) can be used to simultaneously assess variants in potentially responsible genes [15, 16]. MPS of protein coding regions of genes (exomes) or large panels of genes may replace traditional diagnostic testing for tumor markers and follow-up sequencing of candidate genes because of an increased ability to find pathogenic variants. Many centers are turning to MPS, including both panel and exome sequencing (ES), to identify Lynch syndrome and other forms of Mendelian CRC risk. To determine the economic value of replacing traditional diagnostic testing with MPS testing of CRC risk, the benefits of this technology must be considered; these may extend beyond clinical outcomes to patient knowledge of the underlying cause of disease.

While clinical interventions exist for CRC and CRC risk, patients may also value knowledge of the genetic cause of an existing disorder even in the absence of change in management, which is referred to as personal utility [17]. This utility may be partially offset by direct and indirect costs of genetic testing, including adverse impacts of testing. Past studies eliciting preferences for genetic testing in the context of CRC focused on the general population, who consider personal utility and trade-offs differently from patients when valuing testing [4, 18]. Patients’ personal utility for information on genetic causes of CRC and preferences for MPS testing of Mendelian CRC risk are currently unknown.

Our study aims to enumerate patients’ preference-based personal utility for MPS testing of Mendelian CRC risk. Our setting is the New Exome Technology in (NEXT) Medicine Study, a pragmatic randomized control trial in Seattle, Washington, comparing the outcomes of ES relative to traditional diagnostic testing for inherited CRC [19]. We quantify patients’ personal utility for MPS and estimate the monetary value of a MPS test that better identifies genetic causes of CRC using a discrete choice experiment (DCE).

## Materials and methods

DCEs assume that technologies can be described by their characteristics (called attributes), from which individuals derive utility [20]. The attributes are specified across a range of levels. To determine the relative importance of each attribute, DCEs rely on subject responses in scenarios involving two or more competing alternatives, termed choice tasks, which vary based on different combinations of attribute levels. In this way, subjects make trade-offs between risks, benefit, and cost. Discrete choice methods assume that individuals’ choices are representative of their underlying preferences and the values that inform them.

DCE participants included patients registered in the NEXT Medicine Study from 2012 to 2016 who also consented to complete the online questionnaire. All patients in the study had a personal and/or family history of colon cancer and/or polyposis or other features of Lynch syndrome (e.g., endometrial cancer), which resulted in a referral to the University of Washington Genetic Medicine Clinic for usual care genetic testing for Mendelian CRC risk [19]. Usual care testing ordered varied from traditional diagnostic testing for tumor markers to MPS gene panels. Participants with a history of genetic testing for colon cancer or polyps and those with a high probability that their condition was due to one specific gene, such as many hundreds of polyps indicating familial polyposis due to APC gene pathogenic variants, were excluded from the study.

**Fig. 1:**
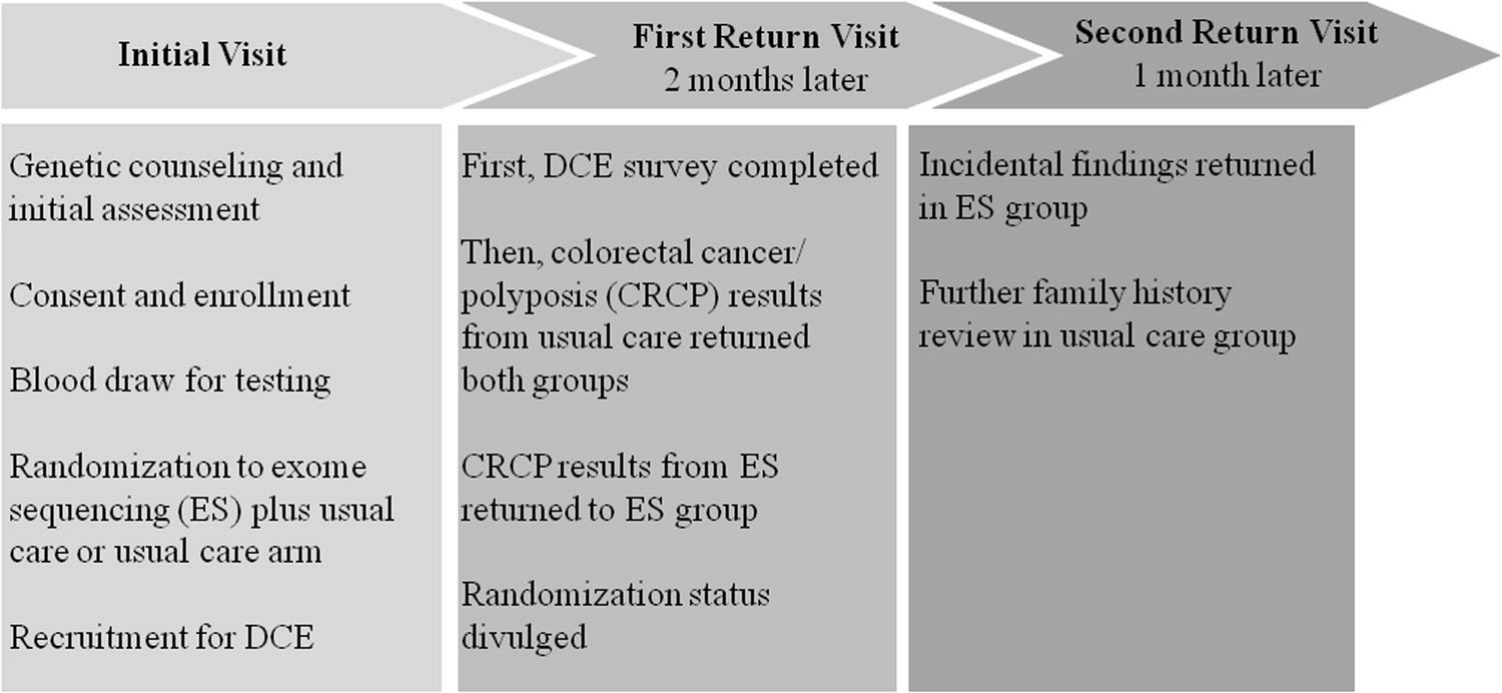
NEXT Medicine Study process for all referred patients with personal and/or family history of colon cancer and/or polyposis or other features of Lynch syndrome

After a genetic counseling session, patients who enrolled in the NEXT Medicine Study provided a blood sample and were randomly assigned to one of two treatment arms for diagnostic testing: a usual care arm or an ES plus usual care arm. In the usual care arm, patients initially underwent traditional diagnostic testing for tumor markers using IHC and/or MSI, followed by a conclusive germline sequencing test if a protein deficiency was detected. Owing to the pragmatic nature of the study, patients in the usual care arm eventually switched from undergoing IHC and/or MSI to receiving a CRC risk gene panel (ColoSeq, University of Washington) [21]. In the ES plus usual care arm, patients also underwent ES to assess the presence of pathogenic variants in all genes potentially responsible for CRC. Figure 1 describes the process and timeline for all patients referred to the NEXT Medicine Study.

We asked participants to respond to a series of 16 choice tasks in a DCE questionnaire. Participants completed a questionnaire approximately two weeks after their first return visit in the NEXT Medicine study, at which time they also received their genetic test results. Following completion of the DCE, we analyzed participant responses and estimated their preferences between alternatives, their personal utility for genetic testing, and WTP for MPS testing of Mendelian CRC risk. Participant responses in this DCE had no impact on genetic tests offered to patients or their clinical care. This study was approved by the University of Washington Research Ethics Board and the BC Cancer Agency Research Ethics Board.

### Development of questionnaire

Questionnaire development has been described previously [22]. Briefly, we began by identifying attributes associated with the benefit that individuals ascribe to genetic testing for inherited CRC. Literature review was used to determine an initial list of factors that may influence preferences, including: test effectiveness, risk of disease, type of test result returned, health consequences, convenience of testing procedure, doctor recommendation to undergo genetic testing, time waiting for results, and cost. Consultation with experts, focus groups with patients who underwent clinical workup for Mendelian CRC risk, and cognitive interviews with patients were undertaken to determine our final list of attributes and levels. We selected the levels of each attribute to accommodate a range of estimates that might be realized for MPS or traditional diagnostic testing.

The final DCE questionnaire incorporated four attributes of varying levels: (1) the proportion of individuals tested who have a genetic cause of their CRC and receive a definitive diagnosis (40%, 60%, 80%, or 90% of individuals tested), also known as the detection rate; (2) wait time for results of all genetic tests (3 weeks, 1.5 months, 3 months, or 6 months); (3) number of tests used to search for a genetic cause of CRC (1 test, 2 tests, 4 tests, or 5 tests); and (4) cost ($425, $1000, $1900, or $2550). Cost was included as an attribute to allow for estimation of WTP.

**Fig. 2:**
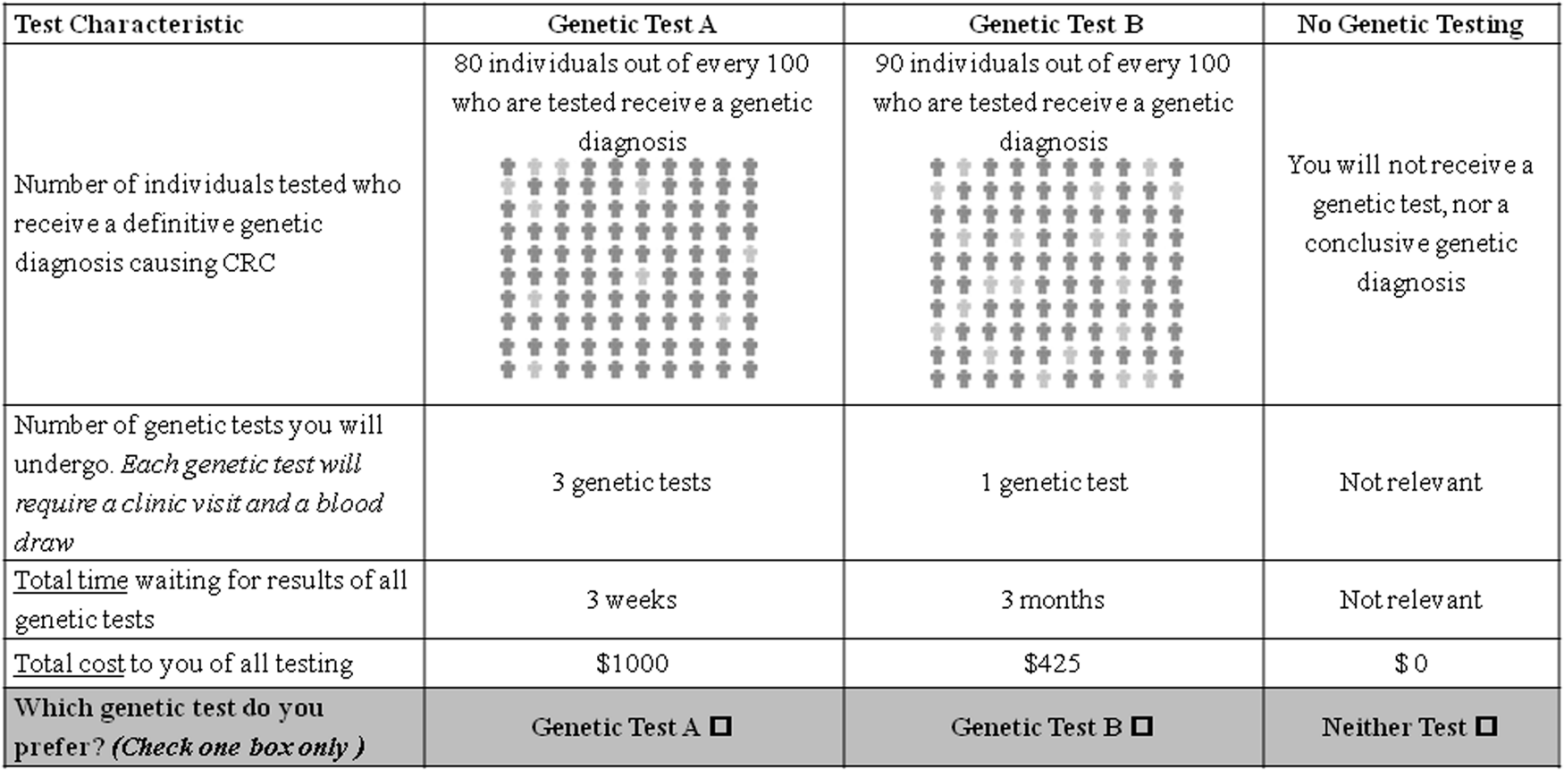
Example of choice task question offered to participants

Each choice task included a choice between two genetic test alternatives and an additional “no test” option, which allowed for patients who did not want to receive genetic testing. We asked respondents to choose their preferred option in each choice task. Figure 2 depicts an example of a choice task. We applied D-optimal procedures to generate a statistically efficient choice-based experimental design using the SAS %ChoicEff macro [23, 24]. This design approach generated 32 choice tasks, which enabled independent and statistically efficient estimation of all main-effects attributes [25]. To reduce respondent burden, the 32 choice tasks were blocked into two questionnaires of 16 tasks. Blocks were orthogonal to the attribute levels to ensure that parameter estimates were independent of each block. Each questionnaire began with an educational component explaining the attributes and attribute levels. A copy of the education section and DCE questionnaires are available in Supplementary Materials and Methods.

### Statistical analysis

We used an error-component mixed logit model to analyze patient responses and estimate attribute coefficients [26]. Coefficient estimates from this model are interpreted as the marginal utility associated with each attribute level, termed part-worth utilities, and can be summed to indicate the overall utility of a good. The model incorporates preference heterogeneity by allowing estimated coefficients to vary across individuals for each attribute level according to a pre-specified distribution.

We specified preferences to be normally distributed across individuals for the following attributes: the proportion of individuals tested who have a Mendelian CRC risk pathogenic or likely pathogenic variant detected (receive a genetic diagnosis), wait time for results of all genetic tests, and number of tests used to search for a Mendelian cause of CRC risk. This assumption allowed participants to have positive or negative preferences for receiving information about the Mendelian causes of their CRC risk. We estimated mean and standard deviation parameters characterizing the distribution of individual preferences in the sampled population. Each parameter estimate had an associated standard error. Wait time for results, the number of genetic tests required, and cost were coded as continuous variables. We used effects coding for the proportion of individuals with a variant detected. We assumed that preferences for cost and for the “no test” option were fixed across individuals. In sensitivity analysis, we included interaction terms in our model to explore differences in preferences across patients with and without a personal history of cancer. We identified statistical significance using a threshold of *p* < 0.05.

Using mean part-worth utility estimates from our final model, we determined the relative importance of each attribute on patients’ choice by comparing the range in estimated utility between best and worst attribute levels, divided by the sum of the ranges of all attributes. We also examined WTP for several scenarios involving testing of Mendelian CRC risk and estimated predicted uptake of each scenario. Scenarios were developed based on expert consultation and were informed by published estimates for currently recommended multistep diagnostic testing and MPS testing for inherited CRC. We assumed that traditional diagnostic testing would identify 40% of individuals with a Mendelian form of CRC risk. We also assumed that traditional diagnostic testing would require three tests resulting in a 3-month wait time for results. Given the sequential nature of traditional diagnostic testing for Mendelian CRC risk at the time the study began, the number of tests and wait time for results could vary considerably across patients.

For the first scenario, we assumed that MPS would identify 60% of patients with a Mendelian form of CRC risk, require 1 test, and involve a 3-week wait time for results. In our second scenario, we assumed that MPS would identify twice as many individuals with a Mendelian form of CRC risk as traditional diagnostic testing (80% of individuals), require 1 test, and involve a 3-week wait time [15]. In our third scenario, we assumed that MPS would identify 90% of individuals, require 1 test, and involve a 1.5-month wait time. For each scenario, we calculated WTP for MPS instead of traditional diagnostic testing using the compensating variation formula [27]. Predicted uptake was based on estimating the percentage of the population predicted to choose a particular scenario [20]. We used the delta method to generate 95% confidence intervals (CIs) for these estimates. All analysis was conducted in Stata 13 [28].

## Results

Out of the 184 participants who were enrolled in the NEXT Medicine study, 122 completed the DCE, resulting in a response rate of 66%. Table 1 summarizes the demographic, socio-economic, and clinical characteristics of patients who did and did not participate in the DCE. The median age of DCE participants was 55 years (interquartile range: 44, 61) and 47% of participants were male. Most participants had completed some form of higher education: 24% had a professional certificate or graduate degree and 66% had a college degree or vocational training. The majority of participants had an annual household income greater than $50,000 (71%), were currently employed (65%), and lived in households of 1 to 2 people (64%). Many participants had a personal history of disease: 36% were diagnosed with CRC, 85% were diagnosed with polyps, and 5% were diagnosed with ovarian or endometrial cancer. While participants’ family history was not always known, 72% had a known family history of CRC or polyposis, 16% had a family history of polyps, and 3% had a history of ovarian or endometrial cancer. We did not detect any significant differences in means or distributions of study characteristics across patients who did and did not participate in the DCE (*p* > 0.05).

**Table 1:** Characteristics of NEXT Medicine study cohort

Characteristics	No. (%) of patients who participated in DCE (*n* = 122)	No. (%) of patients who did not participate in DCE (*n* = 62)
Age, year, median (IQR)	55 (44–61)	50 (42–62)
Sex, male	57 (46.72)	34 (54.84)
Educational background		
Professional or graduate	29 (23.77)	13 (20.97)
College/Vocational	80 (65.57)	38 (61.29)
High school or less	13 (10.66)	11 (17.74)
Annual household income		
<$25,000	12 (9.84)	8 (12.90)
$25,000–$49,999	14 (11.48)	13 (20.97)
$50,000–$100,000	40 (32.79)	12 (19.35)
>$100,000	47 (38.52)	24 (38.71)
Refused/unknown	9 (7.38)	5 (8.06)
Employment status		
Employed	79 (64.75)	41 (66.13)
Unemployed	41 (33.61)	21 (33.87)
Refused/unknown	2 (1.64)	0 (0.00)
Household size		
1 Person	21 (17.21)	11 (17.74)
2 People	57 (46.72)	20 (32.26)
3 People	21 (17.21)	8 (12.90)
≥ 4 People	23 (18.85)	23 (37.10)
Personal history of CRC		
Yes	44 (36.07)	22 (35.48)
Missing	1 (0.82)	1 (1.61)
Personal history of polyps		
Yes	104 (85.25)	50 (80.65)
Missing	5 (4.10)	3 (4.84)
Personal history of ovarian/ endometrial cancer		
Yes	6 (4.92)	4 (6.45)
Not applicable	57 (46.72)	34 (54.84)
Missing	41 (33.61)	18 (29.03)
Family history of CRC/polyposis		
Yes	88 (72.13)	41 (66.13)
Missing/unknown	3 (2.46)	5 (8.06)
Family history of polyps		
Yes	20 (16.39)	11 (17.74)
Missing/unknown	76 (62.30)	43 (69.35)
Family history of ovarian/ endometrial cancer		
Yes	3 (2.46)	1 (1.61)
Missing/unknown	117 (95.90)	59 (95.16)

Two sided *t*-tests showed no statistically significant differences in means of continuous variables across patients who did and did not participate in DCE, non-parametric Mann–Whitney-*U*-tests showed no statistically significant differences in distributions of continuous variables [43], and chi-square tests showed no statistically significant differences in frequency distributions of categorical variables

*IQR* interquartile range

^*^p <  0.05

On average, participants preferred to undergo genetic tests identifying a higher proportion of individuals with a Mendelian CRC risk pathogenic or likely pathogenic variant detected and involving a shorter wait time for results (Table 2). The effect of number of tests on utility was not statistically significantly different from zero at *p* < 0.05. As expected, higher costs of testing and opting out of genetic testing caused disutility. We observed statistically significant preference heterogeneity for all attributes specified as random (*p* < 0.05), as indicated by the standard deviation estimates. For example, holding all else constant, we predicted that 17% of participants would prefer tests involving a longer waiting time. We also predicted that 45% of individuals would prefer fewer tests be used to search for a genetic cause of CRC. In sensitivity analysis, we did not detect statistically significant differences in preferences across patients with and without a personal history of cancer (*p* > 0.05).

**Table 2:** Regression estimates for part-worth utility

Attribute and level	Part-worth utility, mean	Part-worth utility, SD	Part-worth utility < 0
Proportion of individuals identified			
40/100	−2.29*	2.24*	84.7%
60/100	Reference	-	-
80/100	1.14*	0.89*	10.1%
90/100	1.66*	1.94*	19.6%
Number of tests	0.05	0.40*	45.3%
Total wait time (Months)	−0.15*	0.16*	83.3%
Cost ($)	−0.0011*	-	-
Opt out of testing	−7.02*	-	-
Opt in for testing	0 (assumed)	7.90*	-

Part-worth utilities represent the marginal preference-based utilities associated with each attribute level. A positive mean estimate indicates that, on average, patients expressed positive personal utility for the attribute. A negative estimate indicates that, on average, the attribute caused disutility, or a reduction in well-being. Part-worth utilities can be summed to indicate the overall preference-based utility of a good and the ratio of any two part-worth utility estimates shows the marginal rate of substitution between attributes. The estimated SD characterizes the heterogeneity of individual part-worth preference-based utility values in the sampled population

*SD*  standard deviation

*p  < 0.05

**Fig. 3:**
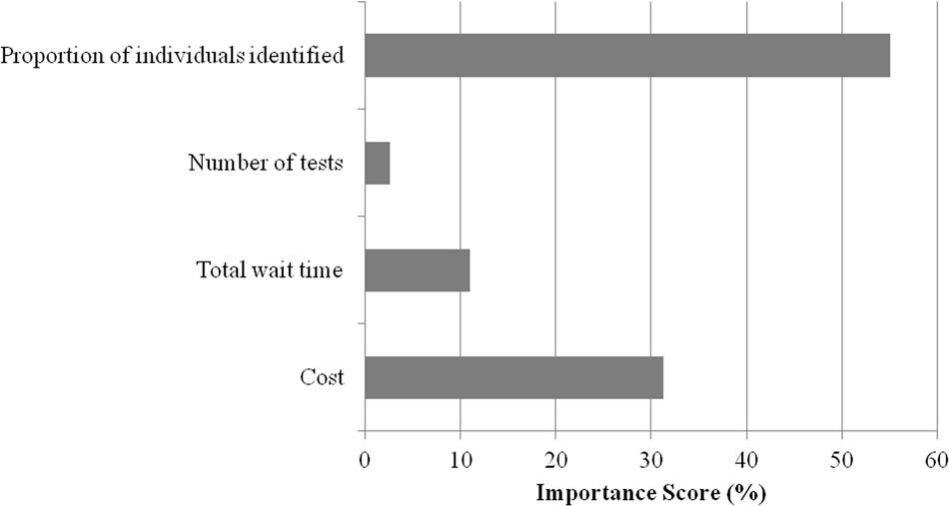
Relative importance of attributes

Figure 3 shows the magnitude and relative importance of each attribute, as measured by importance scores. Importance scores quantify the relative weight an attribute had on patients’ choice. When valuing testing, patients considered the proportion of individuals with a definitive genetic diagnosis identified to be the most important attribute, followed by cost and total wait time. The number of genetic tests required was found to have the least impact on patient preferences.

Table 3 illustrates average WTP estimates and predicted uptake for different tests revealing information about genetic causes of CRC. We assumed that traditional diagnostic testing would identify 40% of individuals with a CRC genetic diagnosis, require 3 tests, and involve 3 months waiting for results. Scenario 1 examined WTP for a MPS test that identified 60% of individuals with a CRC diagnosis, required a single genetic test, and involved a 3-week wait time. The average WTP for this scenario was $400 (95% CI: $300, $500) and 34% of participants were predicted to choose MPS. We predict that 61% of participants would choose not to undergo any genetic testing in this scenario. Scenario 2 examined WTP if MPS identified twice as many individuals with a CRC genetic diagnosis as traditional diagnostic testing, required 1 test, and involved a 3-week wait time for results. The average WTP for this scenario was $1245 (95% CI: $1027, $1462). In this scenario, 73% of participants were predicted to choose MPS and 25% were predicted to choose not to undergo any genetic testing. Scenario 3 examined WTP if MPS identified 90% of individuals with a CRC diagnosis, required one test, and involved a 1.5-month wait time. Under this scenario, average WTP was $1541 (95% CI: $1224, $1859) and 80% of participants were predicted to choose MPS. We predict that 18% would choose not to undergo any genetic testing in scenario 3.

**Table 3:** Willingness-to-pay estimates for genetic testing scenarios

Scenario	New policy scenario where patients choose between two testing options	Prevailing policy scenario	Average incremental WTP, $ (95% CI)	Predicted uptake of new policy scenarios, % (95% CI)
1	MPS Genetic Testing (1) where 60/100 individuals receive a definitive diagnosis, patients undergo 1 test, and spend 3 weeks waiting for results OR traditional diagnostic testing	Traditional diagnostic testing where 40/100 individuals receive a definitive diagnosis, patients undergo 3 tests, and spend 3 months waiting for results	400 (300, 500)	MPS testing (1): 34 (29, 39) Traditional testing: 5 (2, 7)
2	MPS Genetic Testing (2) where 80/100 individuals receive a definitive diagnosis, patients undergo 1 test, and spend 3 weeks waiting for results OR traditional diagnostic testing	Traditional diagnostic testing where 40/100 individuals receive a definitive diagnosis, patients undergo 3 tests, and spend 3 months waiting for results	1245 (1027, 1462)	MPS testing (1): 73 (68, 79) Traditional testing: 2 (0, 3)
3	MPS Genetic Testing (3) where 90/100 individuals receive a definitive diagnosis, patients undergo 1 test, and spend 1.5-months waiting for resultsz OR traditional diagnostic testing	Traditional diagnostic testing where 40/100 individuals receive a definitive diagnosis, patients undergo 3 tests, and spend 3 months waiting for results	1541 (1224, 1859)	MPS testing (2): 80 (74, 87) Traditional testing: 1 (0,3)

*WTP* willingness-to-pay, *CI* confidence interval

## Discussion

We applied a DCE to determine preference-based personal utility for information on Mendelian CRC risk and estimated WTP for MPS testing. We found that, on average, participants preferred to undergo genetic tests detecting a higher proportion of individuals with a definitive genetic etiology and involving a shorter wait time for results. Relative to other attributes, the detection rate of a test had the largest impact on patient preferences. Patient preferences for information on Mendelian CRC risk were heterogeneous. Approximately 17% of participants preferred tests involving a longer waiting time. This heterogeneity is consistent with past literature examining personal utility for genetic information and may be explained by individuals wanting more time to prepare for the results of genetic testing [29–31].

Limited evidence exists on preferences for genetic testing to identify Mendelian CRC risk and our study is the first to explore patients’ preferences for MPS genetic testing. Efforts to date have focused on the general population perspective in the context of population-based screening programs, with researchers concluding that the public is willing to undergo screening, but that choices vary depending on prior experience with genetic testing and anxiety about being genetically predispos1ed to developing CRC [18]. These results align with our findings indicating that while, on average, patients value information on genetic causes of CRC, significant heterogeneity is present in the sample. Previous research finds that attributes related to sensitivity and specificity are among the most important in determining patient and public preferences for population-based CRC screening modalities [32–34]. These results support our findings concerning the impact of the detection rate on patient preferences for MPS genetic testing. Further, our findings regarding the effects of both the detection rate and wait time are consistent with past research exploring preferences for information on genetic causes of diseases [35, 36].

Current guidelines recommend multistep testing for a Mendelian etiology for all patients with newly diagnosed CRC [7, 8]. Our study shows that most patients are willing to pay for information on possible Mendelian causes of CRC and that replacing traditional testing for inherited CRC risk with MPS increases patients’ utility. The extent to which patients valued MPS testing was strongly influenced by the proportion of individuals identified with a genetic CRC diagnosis. Assuming that MPS identifies more individuals with a Mendelian form of CRC risk compared to traditional diagnostic testing, involves fewer genetic tests, and results in a shorter wait time for results, estimated average WTP ranges from $400 (95% CI: $300, $500) to $1541 (95% CI: $1224, $1859). We predict that 34% to 80% of participants would choose to receive MPS over traditional testing and 18% to 61% would choose not to undergo any genetic testing. Similar to past studies, these results suggest that most but not all patients are interested in information about the genetic causes of disease risk [35, 37]. With a view to inform how services can be configured to increase the uptake of testing, further qualitative research is needed exploring respondents’ motivations for forgoing genetic testing.

### Limitations

Our study is subject to some limitations. DCE results depend on the included attributes and levels. Findings may differ if a different set of attributes are used to characterize genetic testing. As recommended by best practice guidance, our patient-centred approach involved selecting attributes and levels that were extensively validated in our population of interest and attributes included in our final DCE were guided by our research question [38]. Discrete choice methods also assume that individuals’ responses in an experiment setting are representative of their true underlying preferences. Given that patient choices in this DCE had no effect on genetic tests offered to patients or their clinical care, our results may be subject to “hypothetical bias”, where stated choices differ from what participants would actually choose. Past research has demonstrated the validity of discrete choice methods and shown that predicted choice probabilities are typically accurate at the aggregate level, if not at the individual level [39]. Hypothetical bias may also be reduced in this context, where all participants were actually undergoing clinical Mendelian CRC risk testing.

Additional limitations of our study involve our respondent characteristics and small sample size. We did not obtain information on respondents’ race or ethnicity, which may influence attitudes toward genetic testing [40]. Respondents in our study were relatively well-educated and had higher socio-economic status than the United States general population [41, 42]. Further, 122 patients completed our DCE, which may affect the precision of estimates and the generalizability of findings. In other jurisdictions, patients with suspected hereditary CRC who are educated and able to afford health-care services are likely to have similar preferences to our study participants. Yet other stakeholders, including health-care professionals and the general public, may value information on genetic causes of CRC differently. Future research examining preferences for MPS in these populations would be beneficial to inform health system planning.

## Conclusion

Patients value information on the Mendelian causes of CRC and many are willing to pay for more effective, efficient genetic testing. Replacing traditional diagnostic testing of Mendelian CRC risk with MPS could increase patients’ perceived welfare. An economic analysis accounting for the full range of costs and benefits of MPS, including the value of knowledge of genetic causes of CRC, is necessary prior to incorporating this technology on a wider scale.

## Electronic supplementary material

Supplemental Material and Methods
