# Supplementary material for: Patient preferences for massively parallel sequencing genetic testing of colorectal cancer risk: a discrete choice experiment
Source: Eur J Hum Genet. 2018 May 25;26(9):1257–65. doi: 10.1038/s41431-018-0161-z (PMC6117311; doi:10.1038/s41431-018-0161-z)
Supplement: Supplementary file 1 — Supplemental Material and Methods [file 41431_2018_161_MOESM1_ESM.docx]

**SUPPLEMENTAL FILE 1: EDUCATION COMPONENT**

| **VALUE OF GENETIC TESTING** |
| --- |

We would like you to value different genetic tests by answering several questions. Each question describes 3 genetic testing scenarios. For each question, we ask you to choose the genetic test you prefer most. Your answers will have no effect on the genetic tests you are offered or your clinical care.

**BACKGROUND INFORMATION**

**Please Read Carefully.** The following description will help you complete the rest of the questionnaire.

The BENEFITS of finding a genetic cause of your disease may include:

- Knowing if the cause of your disease is genetic or environmental
- Better treatment options
- Information regarding your family members’ risk of developing colorectal cancer
- Access for your family to early cancer screening programs

Even the best genetic test available cannot identify all genetic causes. However, there are newer tests that can identify more genetic causes than older genetic tests.

The TRADE-OFFS involved in receiving any genetic test *may* include***:***

- The genetic tests may not be covered by insurance. You may have to pay money for the genetic testing.
- You may need to wait up to 6 months to find out the results of genetic tests.

The following pages describe four characteristics of the genetic tests. These are the characteristics to consider when making a decision about what test you prefer most. **Please consider only these characteristics when making your choices.**

**CHARACTERISTICS OF THE TEST**

The scenarios you will choose between differ according to the following 4 characteristics:

1. **Chance the test will find a gene that caused your cancer**

- - For example, for every 100 people who undergo genetic testing, 80 people who have a genetic cause of their colorectal cancer/ polyposis syndrome will receive a diagnosis. The diagram below shows 80 people (blue) who receive a genetic diagnosis.


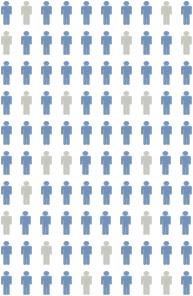


- - Another test may provide a diagnosis in 40 people for every 100 who are tested. The diagram below shows 40 people (blue) who receive a genetic diagnosis related to their disease.

**
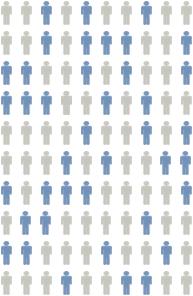
**

1. **Number of genetic tests used to search for a genetic cause of CRC**
   - The number of genetics tests that will be conducted to obtain a diagnosis will be between 1 and 5. The additional tests are required because initial testing found no genetic cause or a genetic cause needs to be confirmed by more testing. *You will need to return to the clinic for the additional testing. Each additional test will require a blood sample.*
2. **Time waiting for the results of all genetic tests**

- It may take **6 weeks** to receive the results of the genetic tests

1. **Total cost to you of all genetic testing**
   - For example, the total cost that you will need to pay out of pocket to have all genetic testing is **$425**

Accurately valuing genetic tests

Please Read.

Before you tell us which test you prefer in each scenario, we want to ask you to help us with a problem we have in studies like this one.

Because this survey doesn’t require people to actually pay for the test, some people do not carefully consider the cost of the test. It may seem easier just to notice that one cost is larger than another cost.

For example, suppse the cost levels are $100, $200, $500, and $750. People think of them as “very low”, “low”, “medium”, and “high”. They don’t really think about what they would have to give up out of their monthly budget – such as restaurant meals or clothes – to pay for the test.

Accurately valuing your preferences for genetic testing is important for health policy. Please help us value the genetic test accurately by paying attention to the actual costs of the test **to you** before deciding which of the alternatives you prefer.

Below is an example question. **Which type of genetic testing would you prefer?** You must choose either Genetic Test A, Test B, or to have no genetic testing. The person who answered the question below decided that Genetic Test B was better than Genetic Test A or No Genetic Testing. They made this decision by comparing Genetic Test A with Genetic Test B, or No Genetic Testing. They stated they were certain of the choice they made.

**Example Question: Which Genetic Test do you prefer?**

| **Test Characteristic** | **Genetic Test A** | **Genetic Test B** | **No Genetic Testing** |
| --- | --- | --- | --- |
| Number of individuals tested who receive a definitive genetic diagnosis causing CRC | 80 individuals out of every 100 who are tested receive a genetic diagnosis  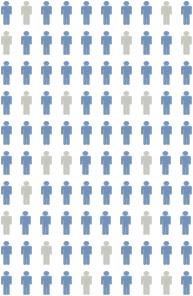 | 90 individuals out of every 100 who are tested receive a genetic diagnosis  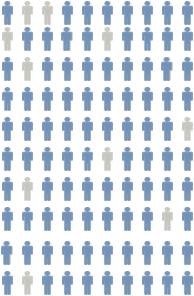 | You will not receive a genetic test, nor a conclusive genetic diagnosis |
| Number of genetic tests you will undergo. *Each genetic test will require a clinic visit and a blood draw* | 3 genetic tests | 1 genetic test | Not relevant |
| Total time waiting for results of all genetic tests | 3 weeks | 3 months | Not relevant |
| Total cost to you of all testing | $1000 | $425 | $ 0 |
| **Which genetic test do you prefer? *(Check one box only )*** | **Genetic Test A 🞎** | **Genetic Test B**  🗹 | **Neither Test 🞎** |

**SUPPLEMENTAL FILE 2: VERSION 1 OF QUESTIONNAIRE**

**Choice Question 1: Which Genetic Test do you prefer?**

| **Test Characteristic** | **Genetic Test A** | **Genetic Test B** | **No Genetic Testing** |
| --- | --- | --- | --- |
| Number of individuals tested who receive a definitive genetic diagnosis causing CRC | 60 individuals out of every 100 who are tested receive a genetic diagnosis  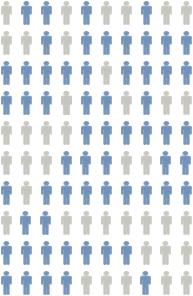 | 40 individuals out of every 100 who are tested receive a genetic diagnosis  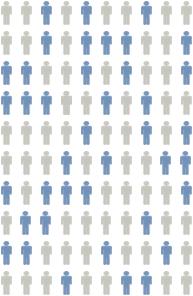 | You will not receive a genetic test, nor a conclusive genetic diagnosis |
| Number of genetic tests you will undergo. *Each genetic test will require a clinic visit and a blood draw* | 1 genetic test | 5 genetic tests | Not relevant |
| Total time waiting for results of all genetic tests | 6 weeks | 3 months | Not relevant |
| Total cost to you of all testing | $2,500 | $1,900 | $ 0 |
| **Which genetic test do you prefer? *(Check one box only )*** | **Genetic Test A 🞎** | **Genetic Test B**  **🞎** | **Neither Test 🞎** |

**Choice Question 2: Which Genetic Test do you prefer?**

| **Test Characteristic** | **Genetic Test A** | **Genetic Test B** | **No Genetic Testing** |
| --- | --- | --- | --- |
| Number of individuals tested who receive a definitive genetic diagnosis causing CRC | 90 individuals out of every 100 who are tested receive a genetic diagnosis  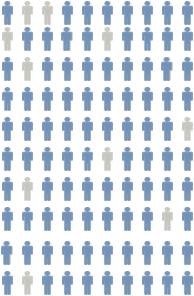 | 40 individuals out of every 100 who are tested receive a genetic diagnosis  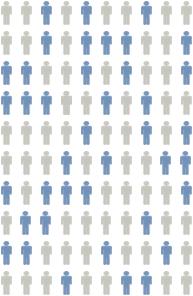 | You will not receive a genetic test, nor a conclusive genetic diagnosis |
| Number of genetic tests you will undergo. *Each genetic test will require a clinic visit and a blood draw* | 2 genetic tests | 1 genetic test | Not relevant |
| Total time waiting for results of all genetic tests | 6 weeks | 6 months | Not relevant |
| Total cost to you of all testing | $1,000 | $2,550 | $ 0 |
| **Which genetic test do you prefer? *(Check one box only )*** | **Genetic Test A 🞎** | **Genetic Test B**  **🞎** | **Neither Test 🞎** |

**Choice Question 3: Which Genetic Test do you prefer?**

| **Test Characteristic** | **Genetic Test A** | **Genetic Test B** | **No Genetic Testing** |
| --- | --- | --- | --- |
| Number of individuals tested who receive a definitive genetic diagnosis causing CRC | 90 individuals out of every 100 who are tested receive a genetic diagnosis  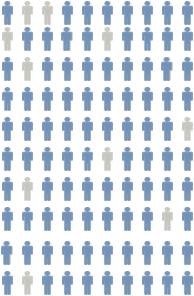 | 60 individuals out of every 100 who are tested receive a genetic diagnosis  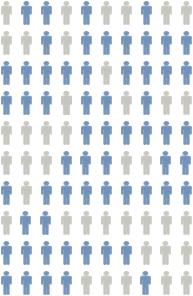 | You will not receive a genetic test, nor a conclusive genetic diagnosis |
| Number of genetic tests you will undergo. *Each genetic test will require a clinic visit and a blood draw* | 2 genetic tests | 4 genetic tests | Not relevant |
| Total time waiting for results of all genetic tests | 3 weeks | 3 months | Not relevant |
| Total cost to you of all testing | $2,550 | $425 | $ 0 |
| **Which genetic test do you prefer? *(Check one box only )*** | **Genetic Test A 🞎** | **Genetic Test B**  **🞎** | **Neither Test 🞎** |

**Choice Question 4: Which Genetic Test do you prefer?**

| **Test Characteristic** | **Genetic Test A** | **Genetic Test B** | **No Genetic Testing** |
| --- | --- | --- | --- |
| Number of individuals tested who receive a definitive genetic diagnosis causing CRC | 90 individuals out of every 100 who are tested receive a genetic diagnosis  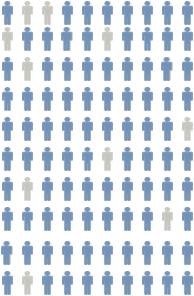 | 80 individuals out of every 100 who are tested receive a genetic diagnosis  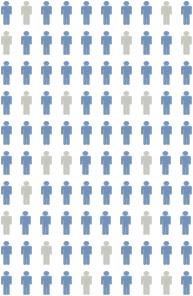 | You will not receive a genetic test, nor a conclusive genetic diagnosis |
| Number of genetic tests you will undergo. *Each genetic test will require a clinic visit and a blood draw* | 1 genetic test | 5 genetic tests | Not relevant |
| Total time waiting for results of all genetic tests | 3 months | 6 weeks | Not relevant |
| Total cost to you of all testing | $1,900 | $425 | $ 0 |
| **Which genetic test do you prefer? *(Check one box only )*** | **Genetic Test A 🞎** | **Genetic Test B**  **🞎** | **Neither Test 🞎** |

**Choice Question 5: Which Genetic Test do you prefer?**

| **Test Characteristic** | **Genetic Test A** | **Genetic Test B** | **No Genetic Testing** |
| --- | --- | --- | --- |
| Number of individuals tested who receive a definitive genetic diagnosis causing CRC | 60 individuals out of every 100 who are tested receive a genetic diagnosis  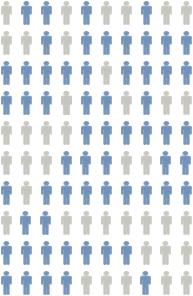 | 40 individuals out of every 100 who are tested receive a genetic diagnosis  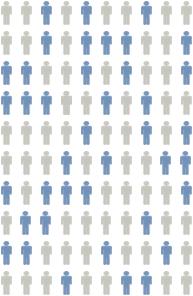 | You will not receive a genetic test, nor a conclusive genetic diagnosis |
| Number of genetic tests you will undergo. *Each genetic test will require a clinic visit and a blood draw* | 5 genetic tests | 4 genetic tests | Not relevant |
| Total time waiting for results of all genetic tests | 6 months | 3 weeks | Not relevant |
| Total cost to you of all testing | $1,000 | $425 | $ 0 |
| **Which genetic test do you prefer? *(Check one box only )*** | **Genetic Test A 🞎** | **Genetic Test B**  **🞎** | **Neither Test 🞎** |

**Choice Question 6: Which Genetic Test do you prefer?**

| **Test Characteristic** | **Genetic Test A** | **Genetic Test B** | **No Genetic Testing** |
| --- | --- | --- | --- |
| Number of individuals tested who receive a definitive genetic diagnosis causing CRC | 80 individuals out of every 100 who are tested receive a genetic diagnosis  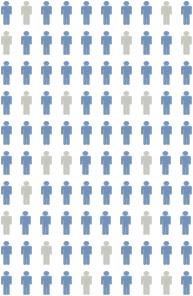 | 40 individuals out of every 100 who are tested receive a genetic diagnosis  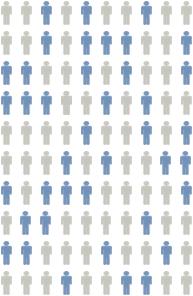 | You will not receive a genetic test, nor a conclusive genetic diagnosis |
| Number of genetic tests you will undergo. *Each genetic test will require a clinic visit and a blood draw* | 4 genetic tests | 5 genetic tests | Not relevant |
| Total time waiting for results of all genetic tests | 3 months | 6 months | Not relevant |
| Total cost to you of all testing | $2,550 | $425 | $ 0 |
| **Which genetic test do you prefer? *(Check one box only )*** | **Genetic Test A 🞎** | **Genetic Test B**  **🞎** | **Neither Test 🞎** |

**Choice Question 7: Which Genetic Test do you prefer?**

| **Test Characteristic** | **Genetic Test A** | **Genetic Test B** | **No Genetic Testing** |
| --- | --- | --- | --- |
| Number of individuals tested who receive a definitive genetic diagnosis causing CRC | 90 individuals out of every 100 who are tested receive a genetic diagnosis  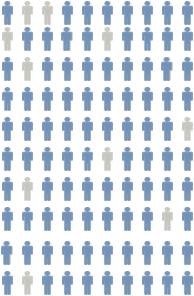 | 80 individuals out of every 100 who are tested receive a genetic diagnosis  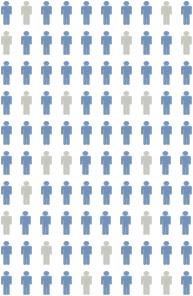 | You will not receive a genetic test, nor a conclusive genetic diagnosis |
| Number of genetic tests you will undergo. *Each genetic test will require a clinic visit and a blood draw* | 4 genetic tests | 1 genetic test | Not relevant |
| Total time waiting for results of all genetic tests | 6 months | 3 months | Not relevant |
| Total cost to you of all testing | $2,550 | $1,000 | $ 0 |
| **Which genetic test do you prefer? *(Check one box only )*** | **Genetic Test A 🞎** | **Genetic Test B**  **🞎** | **Neither Test 🞎** |

**Choice Question 8: Which Genetic Test do you prefer?**

| **Test Characteristic** | **Genetic Test A** | **Genetic Test B** | **No Genetic Testing** |
| --- | --- | --- | --- |
| Number of individuals tested who receive a definitive genetic diagnosis causing CRC | 60 individuals out of every 100 who are tested receive a genetic diagnosis  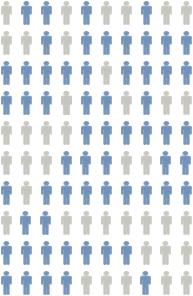 | 80 individuals out of every 100 who are tested receive a genetic diagnosis  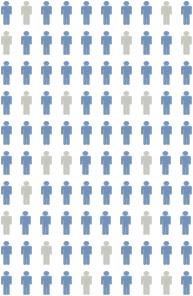 | You will not receive a genetic test, nor a conclusive genetic diagnosis |
| Number of genetic tests you will undergo. *Each genetic test will require a clinic visit and a blood draw* | 2 genetic tests | 5 genetic tests | Not relevant |
| Total time waiting for results of all genetic tests | 6 months | 6 weeks | Not relevant |
| Total cost to you of all testing | $1,000 | $2,550 | $ 0 |
| **Which genetic test do you prefer? *(Check one box only )*** | **Genetic Test A 🞎** | **Genetic Test B**  **🞎** | **Neither Test 🞎** |

**Choice Question 9: Which Genetic Test do you prefer?**

| **Test Characteristic** | **Genetic Test A** | **Genetic Test B** | **No Genetic Testing** |
| --- | --- | --- | --- |
| Number of individuals tested who receive a definitive genetic diagnosis causing CRC | 90 individuals out of every 100 who are tested receive a genetic diagnosis  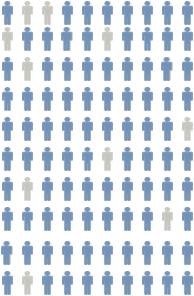 | 40 individuals out of every 100 who are tested receive a genetic diagnosis  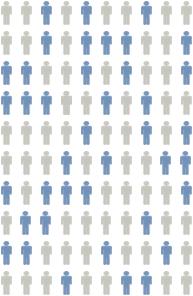 | You will not receive a genetic test, nor a conclusive genetic diagnosis |
| Number of genetic tests you will undergo. *Each genetic test will require a clinic visit and a blood draw* | 1 genetic test | 2 genetic tests | Not relevant |
| Total time waiting for results of all genetic tests | 6 months | 6 weeks | Not relevant |
| Total cost to you of all testing | $425 | $1,900 | $ 0 |
| **Which genetic test do you prefer? *(Check one box only )*** | **Genetic Test A 🞎** | **Genetic Test B**  **🞎** | **Neither Test 🞎** |

**Choice Question 10: Which Genetic Test do you prefer?**

| **Test Characteristic** | **Genetic Test A** | **Genetic Test B** | **No Genetic Testing** |
| --- | --- | --- | --- |
| Number of individuals tested who receive a definitive genetic diagnosis causing CRC | 40 individuals out of every 100 who are tested receive a genetic diagnosis  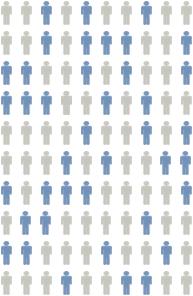 | 80 individuals out of every 100 who are tested receive a genetic diagnosis  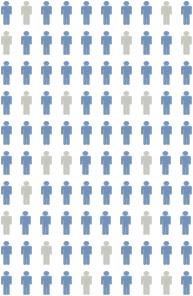 | You will not receive a genetic test, nor a conclusive genetic diagnosis |
| Number of genetic tests you will undergo. *Each genetic test will require a clinic visit and a blood draw* | 4 genetic tests | 2 genetic tests | Not relevant |
| Total time waiting for results of all genetic tests | 6 weeks | 3 weeks | Not relevant |
| Total cost to you of all testing | $1,000 | $425 | $ 0 |
| **Which genetic test do you prefer? *(Check one box only )*** | **Genetic Test A 🞎** | **Genetic Test B**  **🞎** | **Neither Test 🞎** |

**Choice Question 11: Which Genetic Test do you prefer?**

| **Test Characteristic** | **Genetic Test A** | **Genetic Test B** | **No Genetic Testing** |
| --- | --- | --- | --- |
| Number of individuals tested who receive a definitive genetic diagnosis causing CRC | 90 individuals out of every 100 who are tested receive a genetic diagnosis  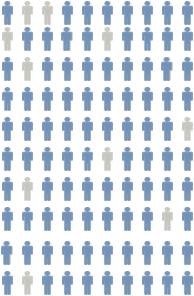 | 60 individuals out of every 100 who are tested receive a genetic diagnosis  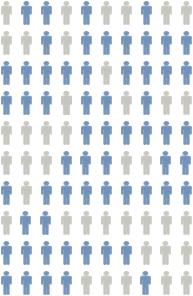 | You will not receive a genetic test, nor a conclusive genetic diagnosis |
| Number of genetic tests you will undergo. *Each genetic test will require a clinic visit and a blood draw* | 1 genetic test | 5 genetic tests | Not relevant |
| Total time waiting for results of all genetic tests | 6 weeks | 3 weeks | Not relevant |
| Total cost to you of all testing | $425 | $2,550 | $ 0 |
| **Which genetic test do you prefer? *(Check one box only )*** | **Genetic Test A 🞎** | **Genetic Test B**  **🞎** | **Neither Test 🞎** |

**Choice Question 12: Which Genetic Test do you prefer?**

| **Test Characteristic** | **Genetic Test A** | **Genetic Test B** | **No Genetic Testing** |
| --- | --- | --- | --- |
| Number of individuals tested who receive a definitive genetic diagnosis causing CRC | 40 individuals out of every 100 who are tested receive a genetic diagnosis  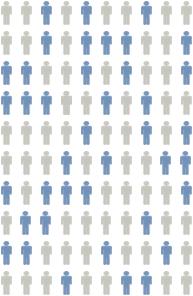 | 90 individuals out of every 100 who are tested receive a genetic diagnosis  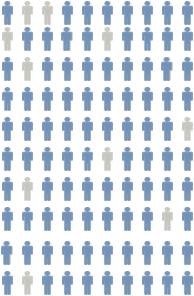 | You will not receive a genetic test, nor a conclusive genetic diagnosis |
| Number of genetic tests you will undergo. *Each genetic test will require a clinic visit and a blood draw* | 1 genetic test | 5 genetic tests | Not relevant |
| Total time waiting for results of all genetic tests | 3 weeks | 3 months | Not relevant |
| Total cost to you of all testing | $1,000 | $1,900 | $ 0 |
| **Which genetic test do you prefer? *(Check one box only )*** | **Genetic Test A 🞎** | **Genetic Test B**  **🞎** | **Neither Test 🞎** |

**Choice Question 13: Which Genetic Test do you prefer?**

| **Test Characteristic** | **Genetic Test A** | **Genetic Test B** | **No Genetic Testing** |
| --- | --- | --- | --- |
| Number of individuals tested who receive a definitive genetic diagnosis causing CRC | 40 individuals out of every 100 who are tested receive a genetic diagnosis  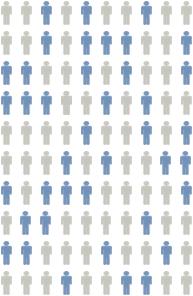 | 60 individuals out of every 100 who are tested receive a genetic diagnosis  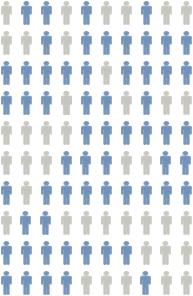 | You will not receive a genetic test, nor a conclusive genetic diagnosis |
| Number of genetic tests you will undergo. *Each genetic test will require a clinic visit and a blood draw* | 2 genetic tests | 4 genetic tests | Not relevant |
| Total time waiting for results of all genetic tests | 3 months | 3 weeks | Not relevant |
| Total cost to you of all testing | $2,550 | $1,900 | $ 0 |
| **Which genetic test do you prefer? *(Check one box only )*** | **Genetic Test A 🞎** | **Genetic Test B**  **🞎** | **Neither Test 🞎** |

**Choice Question 14: Which Genetic Test do you prefer?**

| **Test Characteristic** | **Genetic Test A** | **Genetic Test B** | **No Genetic Testing** |
| --- | --- | --- | --- |
| Number of individuals tested who receive a definitive genetic diagnosis causing CRC | 80 individuals out of every 100 who are tested receive a genetic diagnosis  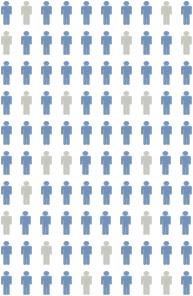 | 60 individuals out of every 100 who are tested receive a genetic diagnosis  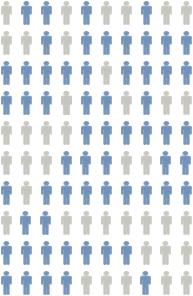 | You will not receive a genetic test, nor a conclusive genetic diagnosis |
| Number of genetic tests you will undergo. *Each genetic test will require a clinic visit and a blood draw* | 4 genetic tests | 1 genetic test | Not relevant |
| Total time waiting for results of all genetic tests | 6 months | 6 weeks | Not relevant |
| Total cost to you of all testing | $1,000 | $1,900 | $ 0 |
| **Which genetic test do you prefer? *(Check one box only )*** | **Genetic Test A 🞎** | **Genetic Test B**  **🞎** | **Neither Test 🞎** |

**Choice Question 15: Which Genetic Test do you prefer?**

| **Test Characteristic** | **Genetic Test A** | **Genetic Test B** | **No Genetic Testing** |
| --- | --- | --- | --- |
| Number of individuals tested who receive a definitive genetic diagnosis causing CRC | 60 individuals out of every 100 who are tested receive a genetic diagnosis  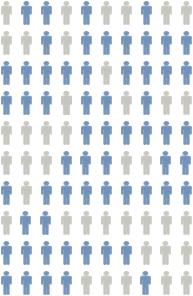 | 80 individuals out of every 100 who are tested receive a genetic diagnosis  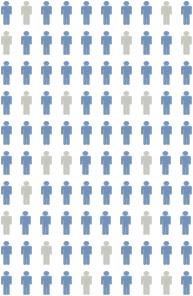 | You will not receive a genetic test, nor a conclusive genetic diagnosis |
| Number of genetic tests you will undergo. *Each genetic test will require a clinic visit and a blood draw* | 2 genetic tests | 1 genetic test | Not relevant |
| Total time waiting for results of all genetic tests | 3 months | 6 months | Not relevant |
| Total cost to you of all testing | $425 | $1,900 | $ 0 |
| **Which genetic test do you prefer? *(Check one box only )*** | **Genetic Test A 🞎** | **Genetic Test B**  **🞎** | **Neither Test 🞎** |

**Choice Question 16: Which Genetic Test do you prefer?**

| **Test Characteristic** | **Genetic Test A** | **Genetic Test B** | **No Genetic Testing** |
| --- | --- | --- | --- |
| Number of individuals tested who receive a definitive genetic diagnosis causing CRC | 90 individuals out of every 100 who are tested receive a genetic diagnosis  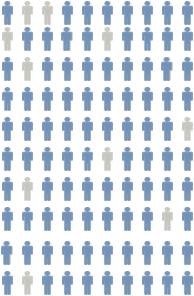 | 80 individuals out of every 100 who are tested receive a genetic diagnosis  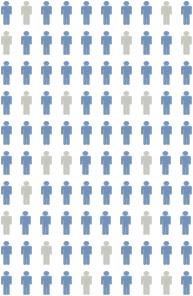 | You will not receive a genetic test, nor a conclusive genetic diagnosis |
| Number of genetic tests you will undergo. *Each genetic test will require a clinic visit and a blood draw* | 5 genetic tests | 2 genetic tests | Not relevant |
| Total time waiting for results of all genetic tests | 3 weeks | 6 months | Not relevant |
| Total cost to you of all testing | $1,000 | $1,900 | $ 0 |
| **Which genetic test do you prefer? *(Check one box only )*** | **Genetic Test A 🞎** | **Genetic Test B**  **🞎** | **Neither Test 🞎** |

**SUPPLEMENTAL FILE 3: VERSION 2 OF QUESTIONNAIRE**

**Choice Question 1: Which Genetic Test do you prefer?**

| **Test Characteristic** | **Genetic Test A** | **Genetic Test B** | **No Genetic Testing** |
| --- | --- | --- | --- |
| Number of individuals tested who receive a definitive genetic diagnosis causing CRC | 40 individuals out of every 100 who are tested receive a genetic diagnosis  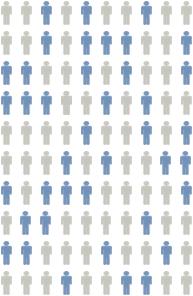 | 90 individuals out of every 100 who are tested receive a genetic diagnosis  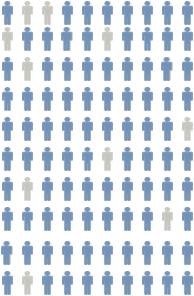 | You will not receive a genetic test, nor a conclusive genetic diagnosis |
| Number of genetic tests you will undergo. *Each genetic test will require a clinic visit and a blood draw* | 1 genetic test | 2 genetic tests | Not relevant |
| Total time waiting for results of all genetic tests | 3 weeks | 6 months | Not relevant |
| Total cost to you of all testing | $425 | $1,000 | $ 0 |
| **Which genetic test do you prefer? *(Check one box only )*** | **Genetic Test A 🞎** | **Genetic Test B**  **🞎** | **Neither Test 🞎** |

**Choice Question 2: Which Genetic Test do you prefer?**

| **Test Characteristic** | **Genetic Test A** | **Genetic Test B** | **No Genetic Testing** |
| --- | --- | --- | --- |
| Number of individuals tested who receive a definitive genetic diagnosis causing CRC | 40 individuals out of every 100 who are tested receive a genetic diagnosis  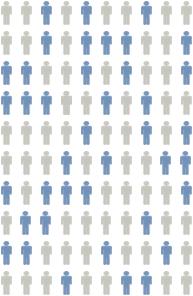 | 90 individuals out of every 100 who are tested receive a genetic diagnosis  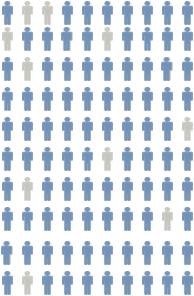 | You will not receive a genetic test, nor a conclusive genetic diagnosis |
| Number of genetic tests you will undergo. *Each genetic test will require a clinic visit and a blood draw* | 2 genetic tests | 4 genetic tests | Not relevant |
| Total time waiting for results of all genetic tests | 6 weeks | 3 weeks | Not relevant |
| Total cost to you of all testing | $1,000 | $1,900 | $ 0 |
| **Which genetic test do you prefer? *(Check one box only )*** | **Genetic Test A 🞎** | **Genetic Test B**  **🞎** | **Neither Test 🞎** |

**Choice Question 3: Which Genetic Test do you prefer?**

| **Test Characteristic** | **Genetic Test A** | **Genetic Test B** | **No Genetic Testing** |
| --- | --- | --- | --- |
| Number of individuals tested who receive a definitive genetic diagnosis causing CRC | 40 individuals out of every 100 who are tested receive a genetic diagnosis  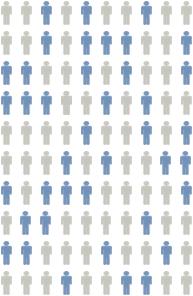 | 90 individuals out of every 100 who are tested receive a genetic diagnosis  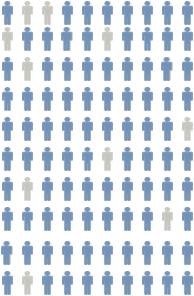 | You will not receive a genetic test, nor a conclusive genetic diagnosis |
| Number of genetic tests you will undergo. *Each genetic test will require a clinic visit and a blood draw* | 4 genetic tests | 5 genetic tests | Not relevant |
| Total time waiting for results of all genetic tests | 3 months | 6 weeks | Not relevant |
| Total cost to you of all testing | $1,900 | $2,550 | $ 0 |
| **Which genetic test do you prefer? *(Check one box only )*** | **Genetic Test A 🞎** | **Genetic Test B**  **🞎** | **Neither Test 🞎** |

**Choice Question 4: Which Genetic Test do you prefer?**

| **Test Characteristic** | **Genetic Test A** | **Genetic Test B** | **No Genetic Testing** |
| --- | --- | --- | --- |
| Number of individuals tested who receive a definitive genetic diagnosis causing CRC | 40 individuals out of every 100 who are tested receive a genetic diagnosis  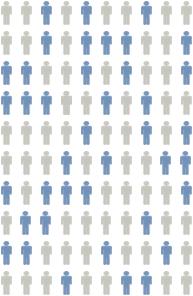 | 90 individuals out of every 100 who are tested receive a genetic diagnosis  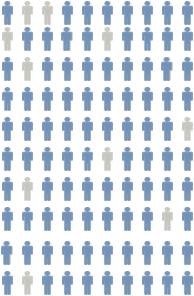 | You will not receive a genetic test, nor a conclusive genetic diagnosis |
| Number of genetic tests you will undergo. *Each genetic test will require a clinic visit and a blood draw* | 5 genetic tests | 1 genetic test | Not relevant |
| Total time waiting for results of all genetic tests | 6 months | 3 months | Not relevant |
| Total cost to you of all testing | $2,550 | $425 | $ 0 |
| **Which genetic test do you prefer? *(Check one box only )*** | **Genetic Test A 🞎** | **Genetic Test B**  **🞎** | **Neither Test 🞎** |

**Choice Question 5: Which Genetic Test do you prefer?**

| **Test Characteristic** | **Genetic Test A** | **Genetic Test B** | **No Genetic Testing** |
| --- | --- | --- | --- |
| Number of individuals tested who receive a definitive genetic diagnosis causing CRC | 60 individuals out of every 100 who are tested receive a genetic diagnosis  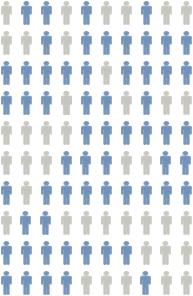 | 40 individuals out of every 100 who are tested receive a genetic diagnosis  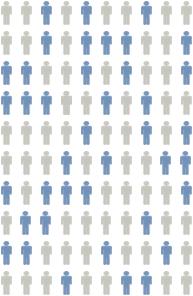 | You will not receive a genetic test, nor a conclusive genetic diagnosis |
| Number of genetic tests you will undergo. *Each genetic test will require a clinic visit and a blood draw* | 1 genetic test | 2 genetic tests | Not relevant |
| Total time waiting for results of all genetic tests | 6 weeks | 3 weeks | Not relevant |
| Total cost to you of all testing | $1,900 | $2,550 | $ 0 |
| **Which genetic test do you prefer? *(Check one box only )*** | **Genetic Test A 🞎** | **Genetic Test B**  **🞎** | **Neither Test 🞎** |

**Choice Question 6: Which Genetic Test do you prefer?**

| **Test Characteristic** | **Genetic Test A** | **Genetic Test B** | **No Genetic Testing** |
| --- | --- | --- | --- |
| Number of individuals tested who receive a definitive genetic diagnosis causing CRC | 60 individuals out of every 100 who are tested receive a genetic diagnosis  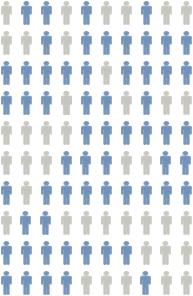 | 40 individuals out of every 100 who are tested receive a genetic diagnosis  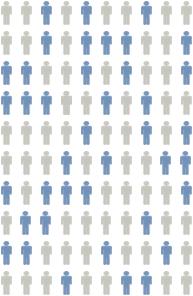 | You will not receive a genetic test, nor a conclusive genetic diagnosis |
| Number of genetic tests you will undergo. *Each genetic test will require a clinic visit and a blood draw* | 2 genetic tests | 4 genetic tests | Not relevant |
| Total time waiting for results of all genetic tests | 3 weeks | 6 months | Not relevant |
| Total cost to you of all testing | $2,550 | $425 | $ 0 |
| **Which genetic test do you prefer? *(Check one box only )*** | **Genetic Test A 🞎** | **Genetic Test B**  **🞎** | **Neither Test 🞎** |

**Choice Question 7: Which Genetic Test do you prefer?**

| **Test Characteristic** | **Genetic Test A** | **Genetic Test B** | **No Genetic Testing** |
| --- | --- | --- | --- |
| Number of individuals tested who receive a definitive genetic diagnosis causing CRC | 60 individuals out of every 100 who are tested receive a genetic diagnosis  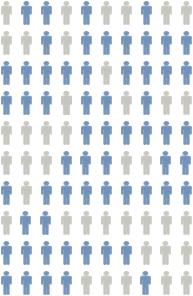 | 40 individuals out of every 100 who are tested receive a genetic diagnosis  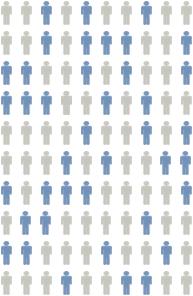 | You will not receive a genetic test, nor a conclusive genetic diagnosis |
| Number of genetic tests you will undergo. *Each genetic test will require a clinic visit and a blood draw* | 4 genetic tests | 5 genetic tests | Not relevant |
| Total time waiting for results of all genetic tests | 6 months | 3 months | Not relevant |
| Total cost to you of all testing | $425 | $1,000 | $ 0 |
| **Which genetic test do you prefer? *(Check one box only )*** | **Genetic Test A 🞎** | **Genetic Test B**  **🞎** | **Neither Test 🞎** |

**Choice Question 8: Which Genetic Test do you prefer?**

| **Test Characteristic** | **Genetic Test A** | **Genetic Test B** | **No Genetic Testing** |
| --- | --- | --- | --- |
| Number of individuals tested who receive a definitive genetic diagnosis causing CRC | 60 individuals out of every 100 who are tested receive a genetic diagnosis  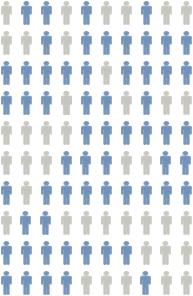 | 40 individuals out of every 100 who are tested receive a genetic diagnosis  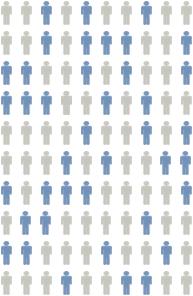 | You will not receive a genetic test, nor a conclusive genetic diagnosis |
| Number of genetic tests you will undergo. *Each genetic test will require a clinic visit and a blood draw* | 5 genetic tests | 1 genetic test | Not relevant |
| Total time waiting for results of all genetic tests | 3 months | 6 weeks | Not relevant |
| Total cost to you of all testing | $1,000 | $1,900 | $ 0 |
| **Which genetic test do you prefer? *(Check one box only )*** | **Genetic Test A 🞎** | **Genetic Test B**  **🞎** | **Neither Test 🞎** |

**Choice Question 9: Which Genetic Test do you prefer?**

| **Test Characteristic** | **Genetic Test A** | **Genetic Test B** | **No Genetic Testing** |
| --- | --- | --- | --- |
| Number of individuals tested who receive a definitive genetic diagnosis causing CRC | 80 individuals out of every 100 who are tested receive a genetic diagnosis  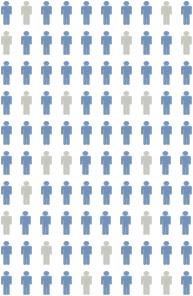 | 60 individuals out of every 100 who are tested receive a genetic diagnosis  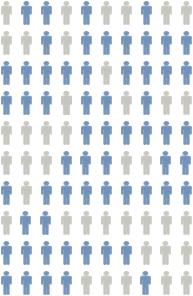 | You will not receive a genetic test, nor a conclusive genetic diagnosis |
| Number of genetic tests you will undergo. *Each genetic test will require a clinic visit and a blood draw* | 1 genetic test | 2 genetic tests | Not relevant |
| Total time waiting for results of all genetic tests | 3 months | 6 weeks | Not relevant |
| Total cost to you of all testing | $2,550 | $425 | $ 0 |
| **Which genetic test do you prefer? *(Check one box only )*** | **Genetic Test A 🞎** | **Genetic Test B**  **🞎** | **Neither Test 🞎** |

**Choice Question 10: Which Genetic Test do you prefer?**

| **Test Characteristic** | **Genetic Test A** | **Genetic Test B** | **No Genetic Testing** |
| --- | --- | --- | --- |
| Number of individuals tested who receive a definitive genetic diagnosis causing CRC | 80 individuals out of every 100 who are tested receive a genetic diagnosis  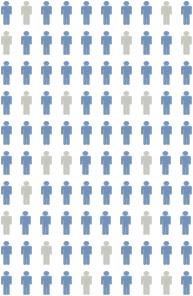 | 60 individuals out of every 100 who are tested receive a genetic diagnosis  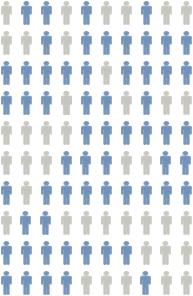 | You will not receive a genetic test, nor a conclusive genetic diagnosis |
| Number of genetic tests you will undergo. *Each genetic test will require a clinic visit and a blood draw* | 2 genetic tests | 4 genetic tests | Not relevant |
| Total time waiting for results of all genetic tests | 6 months | 3 months | Not relevant |
| Total cost to you of all testing | $1,900 | $2,550 | $ 0 |
| **Which genetic test do you prefer? *(Check one box only )*** | **Genetic Test A 🞎** | **Genetic Test B**  **🞎** | **Neither Test 🞎** |

**Choice Question 11: Which Genetic Test do you prefer?**

| **Test Characteristic** | **Genetic Test A** | **Genetic Test B** | **No Genetic Testing** |
| --- | --- | --- | --- |
| Number of individuals tested who receive a definitive genetic diagnosis causing CRC | 80 individuals out of every 100 who are tested receive a genetic diagnosis  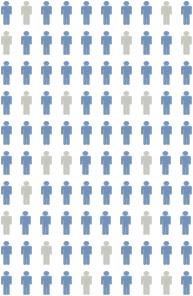 | 60 individuals out of every 100 who are tested receive a genetic diagnosis  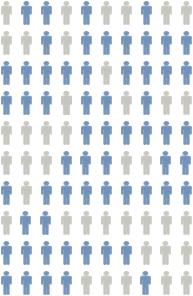 | You will not receive a genetic test, nor a conclusive genetic diagnosis |
| Number of genetic tests you will undergo. *Each genetic test will require a clinic visit and a blood draw* | 4 genetic tests | 5 genetic tests | Not relevant |
| Total time waiting for results of all genetic tests | 3 weeks | 6 months | Not relevant |
| Total cost to you of all testing | $1,000 | $1,900 | $ 0 |
| **Which genetic test do you prefer? *(Check one box only )*** | **Genetic Test A 🞎** | **Genetic Test B**  **🞎** | **Neither Test 🞎** |

**Choice Question 12: Which Genetic Test do you prefer?**

| **Test Characteristic** | **Genetic Test A** | **Genetic Test B** | **No Genetic Testing** |
| --- | --- | --- | --- |
| Number of individuals tested who receive a definitive genetic diagnosis causing CRC | 80 individuals out of every 100 who are tested receive a genetic diagnosis  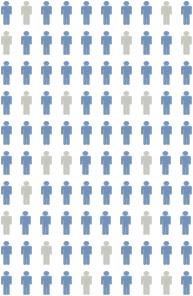 | 60 individuals out of every 100 who are tested receive a genetic diagnosis  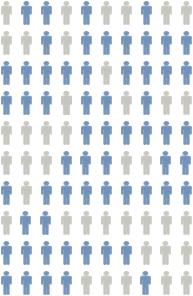 | You will not receive a genetic test, nor a conclusive genetic diagnosis |
| Number of genetic tests you will undergo. *Each genetic test will require a clinic visit and a blood draw* | 5 genetic tests | 1 genetic test | Not relevant |
| Total time waiting for results of all genetic tests | 6 weeks | 3 weeks | Not relevant |
| Total cost to you of all testing | $425 | $1,000 | $ 0 |
| **Which genetic test do you prefer? *(Check one box only )*** | **Genetic Test A 🞎** | **Genetic Test B**  **🞎** | **Neither Test 🞎** |

**Choice Question 13: Which Genetic Test do you prefer?**

| **Test Characteristic** | **Genetic Test A** | **Genetic Test B** | **No Genetic Testing** |
| --- | --- | --- | --- |
| Number of individuals tested who receive a definitive genetic diagnosis causing CRC | 90 individuals out of every 100 who are tested receive a genetic diagnosis  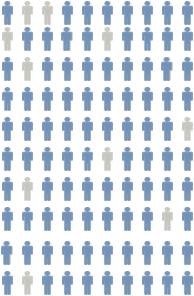 | 80 individuals out of every 100 who are tested receive a genetic diagnosis  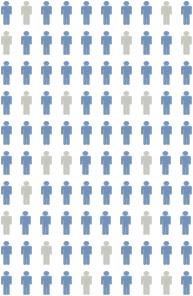 | You will not receive a genetic test, nor a conclusive genetic diagnosis |
| Number of genetic tests you will undergo. *Each genetic test will require a clinic visit and a blood draw* | 1 genetic test | 2 genetic tests | Not relevant |
| Total time waiting for results of all genetic tests | 6 months | 3 months | Not relevant |
| Total cost to you of all testing | $1,000 | $1,900 | $ 0 |
| **Which genetic test do you prefer? *(Check one box only )*** | **Genetic Test A 🞎** | **Genetic Test B**  **🞎** | **Neither Test 🞎** |

**Choice Question 14: Which Genetic Test do you prefer?**

| **Test Characteristic** | **Genetic Test A** | **Genetic Test B** | **No Genetic Testing** |
| --- | --- | --- | --- |
| Number of individuals tested who receive a definitive genetic diagnosis causing CRC | 90 individuals out of every 100 who are tested receive a genetic diagnosis  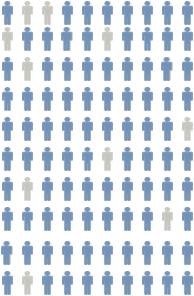 | 80 individuals out of every 100 who are tested receive a genetic diagnosis  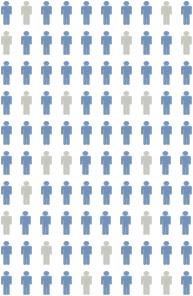 | You will not receive a genetic test, nor a conclusive genetic diagnosis |
| Number of genetic tests you will undergo. *Each genetic test will require a clinic visit and a blood draw* | 2 genetic tests | 4 genetic tests | Not relevant |
| Total time waiting for results of all genetic tests | 3 months | 6 weeks | Not relevant |
| Total cost to you of all testing | $425 | $1,000 | $ 0 |
| **Which genetic test do you prefer? *(Check one box only )*** | **Genetic Test A 🞎** | **Genetic Test B**  **🞎** | **Neither Test 🞎** |

**Choice Question 15: Which Genetic Test do you prefer?**

| **Test Characteristic** | **Genetic Test A** | **Genetic Test B** | **No Genetic Testing** |
| --- | --- | --- | --- |
| Number of individuals tested who receive a definitive genetic diagnosis causing CRC | 90 individuals out of every 100 who are tested receive a genetic diagnosis  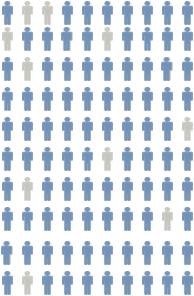 | 80 individuals out of every 100 who are tested receive a genetic diagnosis  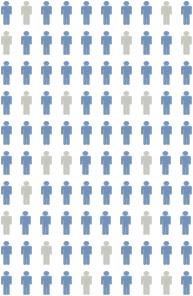 | You will not receive a genetic test, nor a conclusive genetic diagnosis |
| Number of genetic tests you will undergo. *Each genetic test will require a clinic visit and a blood draw* | 4 genetic tests | 5 genetic tests | Not relevant |
| Total time waiting for results of all genetic tests | 6 weeks | 3 weeks | Not relevant |
| Total cost to you of all testing | $2,550 | $425 | $ 0 |
| **Which genetic test do you prefer? *(Check one box only )*** | **Genetic Test A 🞎** | **Genetic Test B**  **🞎** | **Neither Test 🞎** |

**Choice Question 16: Which Genetic Test do you prefer?**

| **Test Characteristic** | **Genetic Test A** | **Genetic Test B** | **No Genetic Testing** |
| --- | --- | --- | --- |
| Number of individuals tested who receive a definitive genetic diagnosis causing CRC | 90 individuals out of every 100 who are tested receive a genetic diagnosis  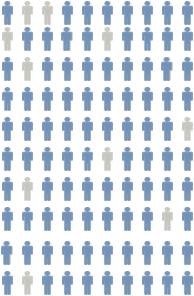 | 80 individuals out of every 100 who are tested receive a genetic diagnosis  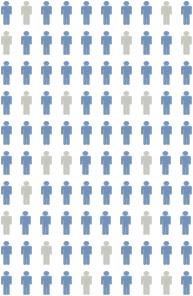 | You will not receive a genetic test, nor a conclusive genetic diagnosis |
| Number of genetic tests you will undergo. *Each genetic test will require a clinic visit and a blood draw* | 5 genetic tests | 1 genetic test | Not relevant |
| Total time waiting for results of all genetic tests | 3 weeks | 6 months | Not relevant |
| Total cost to you of all testing | $1,900 | $2,550 | $ 0 |
| **Which genetic test do you prefer? *(Check one box only )*** | **Genetic Test A 🞎** | **Genetic Test B**  **🞎** | **Neither Test 🞎** |
